# Epidemiology and Treatment of Olecranon Fractures: a nationwide register-based analysis of 27,880 cases in Denmark from 1999 to 2018

**DOI:** 10.1186/s13018-025-05970-2

**Published:** 2025-06-03

**Authors:** Walid Zeyghami, Dennis Karimi, William M. Hagemann, Per H. Gundtoft, Bjarke Viberg, Tazio Maleitzke

**Affiliations:** 1 Department of Orthopaedic Surgery, Trauma Orthopaedic Research Copenhagen Hvidovre (TORCH), Copenhagen University Hospital - Amager and Hvidovre, Hvidovre, Denmark; 2 Department of Orthopaedic Surgery, Copenhagen University Hospital - Zealand University Hospital Køge, Køge, Denmark; 3 Department of Orthopaedic Surgery and Traumatology, Lillebaelt Hospital Kolding, University Hospital of Southern Denmark, Kolding, Denmark; 4 Department of Orthopaedic Surgery and Traumatology, Aarhus University Hospital, Aarhus, Denmark; 5 Department of Orthopaedic Surgery and Traumatology, Odense University Hospital, Odense, Denmark; 6 Department of Clinical Medicine, University of Copenhagen, Copenhagen, Denmark; 7 Charité – Universitätsmedizin Berlin, Corporate Member of Freie Universität Berlin and Humboldt-Universität zu Berlin, Center for Musculoskeletal Surgery, Berlin, Germany; 8 Berlin Institute of Health at Charité – Universitätsmedizin Berlin, Julius Wolff Institute, Berlin, Germany

**Keywords:** Elbow fracture, Tension-band wiring, Plate fixation, Non-surgical treatment, Conservative treatment

## Abstract

**Background:**

Olecranon fractures (OFs) account for approximately 20% of proximal forearm fractures. Displaced or unstable OFs are typically treated surgically with tension-band wiring (TBW) or plate fixation (PF). While comparative works on surgical OF management exist, epidemiological studies are limited by short time spans and small sample sizes. This study investigates OF incidence rates (IRs), and treatment trends in Denmark over a 20-year period from 1999 to 2018.

**Patients and methods:**

Population-based Danish National Patient Register study on OFs in adult patients from 1999 to 2018. Patients ≥ 20 years diagnosed with OF (ICD-10: S520) were included. Age, sex, and treatment were recorded. Treatment was classified as surgical if relevant surgical procedure codes were recorded within 21 days of OF diagnosis. In the absence of such codes, treatment was classified as non-surgical.

**Results:**

A total of 27,880 OF cases (61% female) were identified between 1999 and 2018. The overall mean IR was 33/100,000/year, increasing from 31 in 1999 to 40 in 2018. Females and males had similar IRs between 20 and 49 years, while females ≥ 50 years showed markedly higher IRs than males. Non-surgical treatment was predominant (67%, range: 64–72%). Surgical treatment was more frequent in females (36%, range: 30–42%) than in males (28%, range: 25–34%) and more frequent in patients ≥ 50 years (37%, range: 32–41%) than in patients < 50 years (24%, range: 22–26). Interestingly, from 2013 onwards, surgical treatment decreased in patients ≥ 70 years. Over time, PF use increased from 7% in 1999 to 45% in 2018. TBW declined from 89% in 1999 to 46% in 2018.

**Conclusions:**

The incidence of OFs increased by 29% over the 20-year study period. Non-surgical treatment was predominant across all ages but decreased markedly in older patients. Over the assessed two decades, PF increased and TBW decreased in popularity for surgically managed OFs. Studies identifying which patients may benefit most from surgical and non-surgical treatment by incorporating patient-specific factors will help to refine decision-making and optimize clinical outcomes.

## Background

Olecranon fractures (OFs) account for approximately 20% of all proximal forearm fractures [1, 2]. Displaced and unstable OFs are typically treated surgically with tension-band wiring (TBW) or plate fixation (PF) [3–6], while undisplaced OFs may be treated non-surgically with cast immobilization of the elbow [7, 8].

Although OF management is widely studied [6, 7, 9, 10], only two studies examine the epidemiology of OFs [1, 11]. Both studies are limited by their short time spans, ranging from one to four years, and only one study reports incidence rates [1]. To date, no large-scale, long-term population-based studies have described the incidence or treatment patterns of OFs. Despite the relatively high prevalence of OFs, there is limited high-quality evidence defining optimal treatment strategies. As a result, a variety of treatment approaches are currently used in clinical practice. Large-scale register works are important to study incidences of representative populations as well as current and past treatment practices and how these are distributed across different age groups.

Over time, treatment approaches for OFs have shifted in response to evolving surgical techniques and a better understanding of patient-specific needs. While TBW has historically been the most common surgical method, PF has gained popularity due to enhanced stability and lower risk of hardware-related complications [10, 12–15]. Furthermore, management strategies now increasingly reflect patient age and activity level, with non-surgical approaches considered for elderly patients with low functional demands [6, 16]. These trends emphasize the importance of analyzing long-term treatment patterns to optimize care for diverse patient groups.

This study aimed to evaluate incidence rates (IRs), and treatment trends of OFs in Denmark over two decades, from 1999 to 2018.

## Methods

### Study design

This is a population-based register study on OFs in adult patients from the Danish National Patient Register (DNPR) between 1999 and 2018. Study results are reported according to the RECORD guidelines [17].

### Setting

The study was conducted in Denmark, where all permanent residents are assigned a unique, 10-digit identification number. This enables linkage of patients on an individual level across all Danish medical databases [18]. The Danish National Health Service provides free access to healthcare including access to general practitioners and general hospital care with emergency service for all permanent residents [19]. Private emergency treatment are not available in Denmark, therefore nearly all fracture treatment is conducted at public hospitals.

### Data source

Patient data were extracted from the DNPR covering a period from 1999 to 2018. The DNPR was established in 1977, and is a comprehensive administrative database covering 98.8% of all hospital contacts in Denmark [20]. It is mandatory to report administrative data such as age, sex, diagnosis and procedure codes monthly for all public and private hospitals. Since 1995 diagnosis codes are registered using the International Statistical Classification of Diseases version 10 (ICD-10) and surgical procedure codes according to The Nordic Medico-Statistical Committee (NOMESCO) system [21].

### Participants

The study population included patients ≥ 20 years with an OF diagnosis (ICD-10: S520). The accuracy of the OF diagnosis code in the DNPR has not yet been validated, however Schmidt, et al. reported a positive predictive value of 83% for all orthopedic diagnosis codes registered in the DNPR [20]. The diagnosis codes for humerus fractures, hip fractures and ankle fractures have been independently validated and demonstrated positive predictive values ranging from 78 to 92% [22–24]. Foreign patients without a permanent residence in Denmark have no records of their sex in the DNPR and were thus excluded from the study population.

### Variables

Data on age, sex, diagnosis code, date of diagnosis, procedure code and date of procedure were extracted. Treatments were classified as surgical if relevant surgical procedure codes were recorded within 21 days of the initial OF diagnosis. This time frame was chosen to (i) cover patients receiving surgery primarily, and (ii) those treated conservatively, who were converted to surgery following secondary fracture displacement. In Denmark, patients that are primarily treated conservatively, are usually seen for a 1-week-follow-up where they are screened for secondary displacement and planned for subsequent surgery if required.

In the absence of surgical procedure codes, treatment was classified as non-surgical. The surgical procedure codes were divided into four categories: TBW (KNCJ40), plate fixation (KNCJ60), arthroplasty (KNCB0, KNCB1, KNCB2, KNCB3 and KNCB4), and other procedures (nailing [KNCJ50], external fixation [KNCJ20], combined method [KNCJ80] and unspecified method [KNCJ90]).

### Statistics

Descriptive statistics were used to report age, sex, number of OFs and treatment. We report incidences as cases per 100,000 persons a year, where the population data for each year were obtained from Statistics Denmark [25]. Incidences were reported by age in 10-year intervals: 20–29, 30–39, 40–49, 50–59, 60–69, 70–79, 80–89 and ≥ 90 years. In addition, treatment is reported for patients between 20 and 70 and > 70 years.

### Ethics

DNPR data are based on diagnosis and procedure codes, with all information being anonymized to ensure patient identities remain confidential. According to Danish Law ethical approval is not applicable (Table 1).

**Table 1:** Demographics of olecranon fracture (OF) patients from 1999 to 2018 in Denmark

	Total	1999	2000	2001	2002	2003	2004	2005	2006	2007	2008	2009	2010	2011	2012	2013	2014	2015	2016	2017	2018
N	27880	1271	1287	1186	1260	1204	1114	1299	1246	1205	1294	1273	1449	1525	1337	1599	1597	1506	1700	1736	1792
Fracture incidence per 10^5^	33.1	31.3	31.6	29.1	30.9	29.5	27.3	31.8	30.4	29.3	31.3	30.6	34.6	36.2	31.6	37.5	37.1	34.7	38.7	39.1	40.1
Sex																					
Female	17116	760	739	713	746	677	687	807	814	713	786	790	902	943	858	966	1030	973	1054	1061	1097
Male	10764	511	548	473	514	527	427	492	432	492	508	483	547	582	479	633	567	533	646	675	695
Age																					
20–29	3188	180	201	173	154	162	115	117	116	135	140	146	159	145	158	156	161	184	200	174	212
30–39	2710	168	174	138	164	152	139	147	125	114	116	99	148	162	121	127	96	127	115	127	151
40–49	3438	159	154	148	163	151	134	185	168	161	158	161	181	176	191	207	231	170	175	186	179
50–59	4706	224	204	177	221	203	174	202	205	198	216	207	278	275	223	263	263	231	296	325	321
60–69	5030	163	154	181	151	173	191	195	215	200	235	244	262	317	241	343	333	317	373	366	376
70–79	4438	192	184	177	206	176	169	210	182	191	197	195	196	217	188	242	284	261	302	334	335
80–89	3398	146	172	145	164	151	158	197	189	165	169	189	176	166	168	203	159	163	185	165	168
≥ 90	972	39	44	47	37	36	34	46	46	41	63	32	49	67	47	58	70	53	54	59	50
Median	60	55.8	55.6	57.6	56.7	56.8	62.4	60	60.4	59.8	63.1	61	58.5	60.8	59.8	61.4	64.4	61.3	61.7	61.7	61.5

## Results

**Fig. 1:**
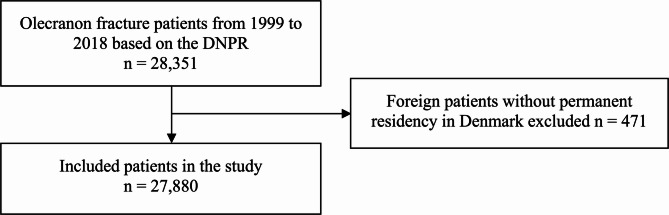
Study population of olecranon fractures based on the Danish National Patient Register (DNPR)

We included 28,351 patients with an OF diagnosis code from 1999 to 2018. A total of 471 foreign patients were excluded, which resulted in a total of 27,880 patients (Fig. 1).

### Epidemiology

**Fig. 2:**
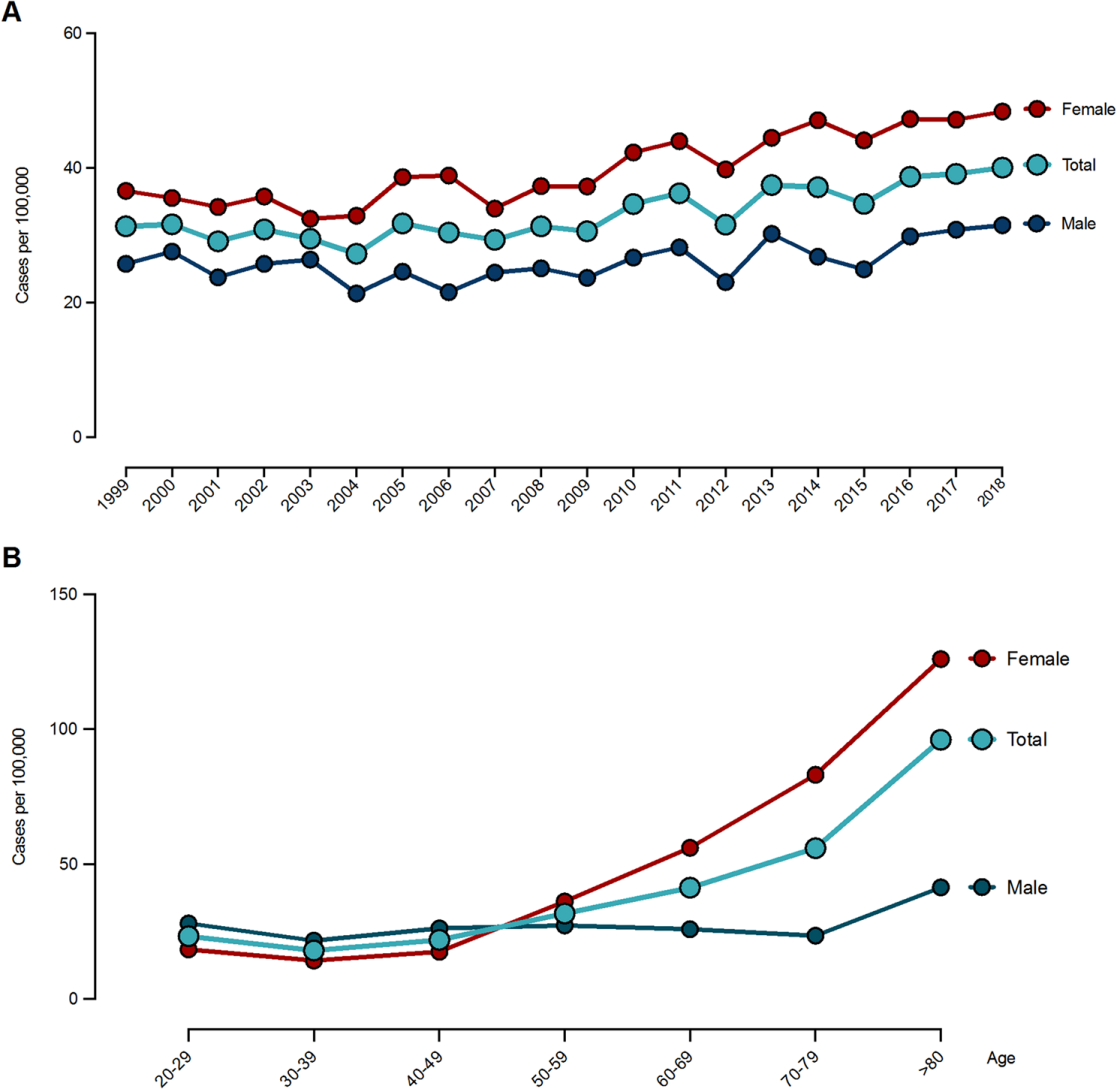
Incidence rates of olecranon fractures from 1999 to 2018 in Demark. Distribution stratified by sex over time (**A**), and according to age groups (**B**)

The mean age was 61 years and 61% of patients were female. The incidence of OFs increased by 29% over the study period from 31/100,000/year in 1999 to 40/100,000/year in 2018. The overall mean incidence was 33/100,000/year (Fig. 2A). Males aged 20–49 had higher IRs than females in the same age group. This changed to predominately females in the ≥ 50 years group (Fig. 2B). Overall, the highest IR was observed among patients ≥ 80. While this was true for males and females, females still had higher IRs than males in this age group (Fig. 2B). The overall and sex-stratified age-specific IR gradually increased with advancing age in females but remained stable in males (Fig. 2B).

### Treatment

**Fig. 3:**
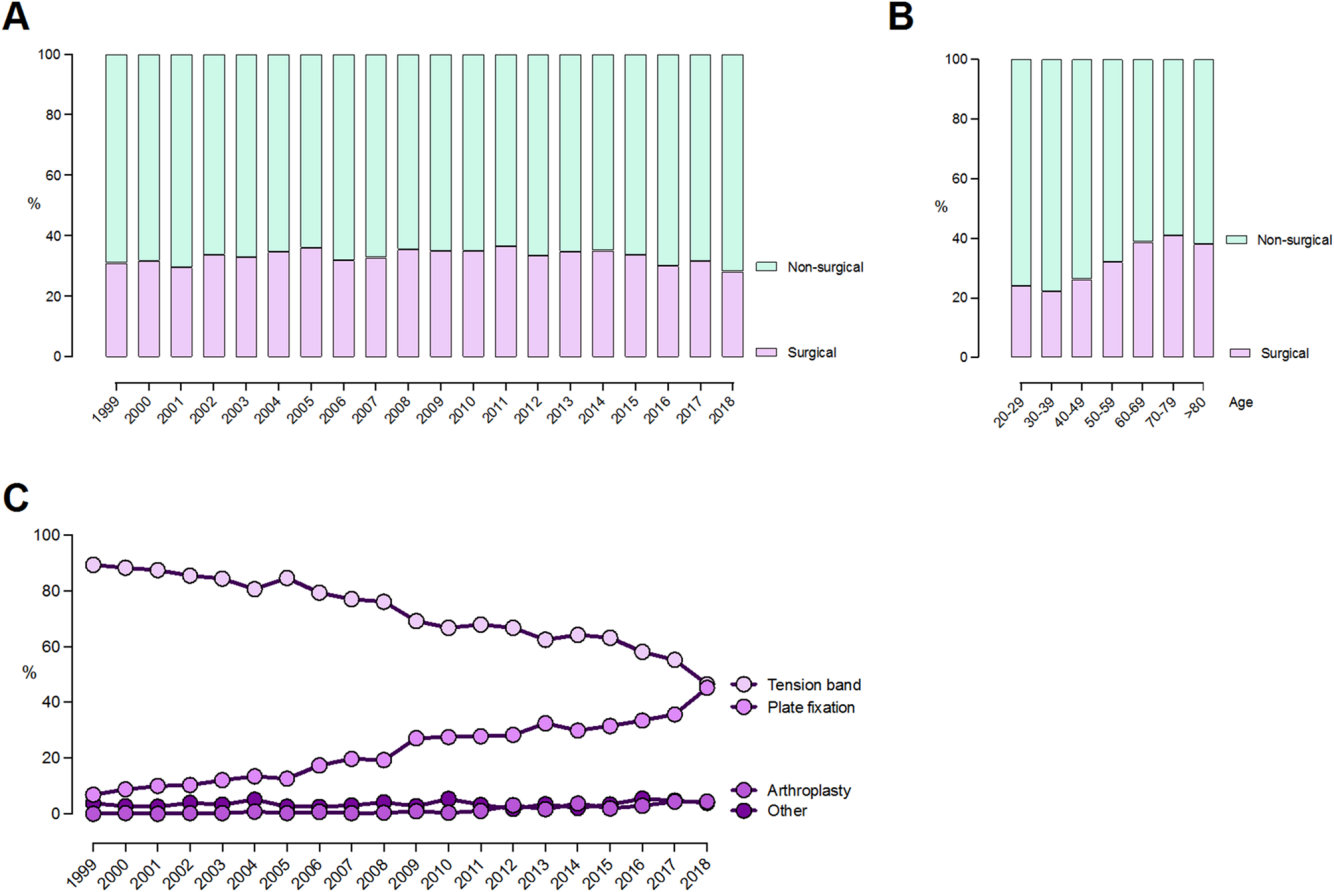
Treatment of olecranon fractures from 1999 to 2018 in Demark. Distribution stratified by surgical or non-surgical treatment over time (**A**), and according to age groups (**B**). Distribution of different surgical treatments over time (**C**)

Overall, 67% of cases were treated non-surgically (range: 64% in 2005 to 72% in 2018, Fig. 3A). This remained stable throughout the study period. Additionally, females were more likely to undergo surgical treatment than males throughout the entire study period with 36% of females undergoing surgery (range: 30–42%) compared to 28% of males (range: 25–34%). Surgical treatment was more frequent in patients ≥ 50 years (37%, range: 32–41%) than in patients < 50 years (24%, range: 22–26) (Fig. 3B).

The distribution of surgical and non-surgical treatment remained stable throughout the study period for patients aged 20–69 years, with 25–33% treated surgically and 67–75% treated non-surgically. In patients aged ≥ 70 years, surgical treatment decreased, and non-surgical treatment increased from 2013 onwards (Fig. 3A, B).

The choice of surgical implants changed considerably over time, with the use of TBW continuously decreasing and the use of PF significantly increasing from 1999 to 2018. In 1999, TBW was utilized in 89% of surgically treated patients, whereas PF was used in only 7% of cases. By 2018, the use of PF had increased to 45%, matching TBW, which was used in 46% of cases. The use of other surgical techniques remained stable throughout the period (Fig. 3C).

## Discussion

In this study, we assessed the epidemiology and treatment of OFs over a 20-year period in Denmark. The IR increased over time from 31 in 1999 to 40 in 2018. While IRs generally increased with age, this was especially relevant in females ≥ 50 years. Most patients were treated non-surgically (67%). Interestingly, surgical treatment became more frequent in patients ≥ 50 years. While most surgically treated patients underwent TBW in 1999, we found an even distribution of TBW and PF in 2018.

Reported IRs of OFs vary widely in the literature, mostly due to differences in study design and fracture classification. Duckworth, et al. reported a much lower IR (12/100,000/year) [1], and Axenhus, et al. a much higher IR (333/100,000/year) [26]. This is likely due to alternating inclusion criteria, time periods and demographic populations. Despite these differences, both studies, like ours, observed a similar increase in IRs among patients with increasing age > 50 years, highlighting rising fracture rates with age.

We found that in the Danish population 67% of patients were treated non-surgically, which remained stable through the study period. With increasing age, people were more likely to be treated surgically. In the 20–29 years group, 24% were treated surgically, while in the 70–79 years group, 41% were operated. While this suggests an increased likelihood of surgical treatment with age, it is important to note that the majority (59%) of elderly patients were still treated non-surgically. A reason for this may be more complex fracture patterns, resulting from osteoporosis and less controlled falls due to higher frailty scores in the elderly [27, 28]. A similar trend was seen in a Swedish observational study [11]. However, since we have no data on fracture patterns, it is difficult to directly compare our findings with the study by Brüggemann et al. [11].

The choice between surgical and non-surgical treatment for displaced OFs remains a topic of debate, particularly in elderly patients [9, 16, 29]. Retrospective cohort data indicate that non-surgical treatment in patients ≥ 70 with displaced OFs provides satisfactory functional range of motion and a high level of patient satisfaction [29]. A randomized controlled trial showed that patients over 75 years who underwent surgery with either TBW or PF experienced a high complication rate of 81.8% compared to 14.3% in those receiving conservative treatment, which led to the trial being prematurely terminated [16].

As a reaction to those results, new guidelines which favor a more conservative approach for OF patients ≥ 75 years were introduced in Denmark in 2018 [30]. We observed a noticeable shift toward more non-surgical treatment in elderly patients from 2013 onwards, potentially driven by study results showing that non-surgical treatment is a viable alternative in elderly patient cohorts [1, 2, 16, 29, 31].

In 1999, TBW was the preferred surgical method to treat OFs. However, a gradual shift toward an increased use of PF was observed from 2002 onwards, with both methods showing parity in 2018. The introduction of locking plates around 2000 may have contributed to this trend [32], which is likely to have continued beyond 2018.

Additionally, multiple studies have highlighted the advantages of PF over TBW, including a lower risk of implant irritation, reduced rates of implant removal, superior biomechanical properties, and enhanced interfragmentary compression [10, 12, 15, 33–35]. Despite these benefits, no significant differences in functional outcomes, such as range of motion or Disabilities of the Arm, Shoulder, and Hand (DASH) score, have been observed comparing the two techniques [15, 33, 34].

In recent years, tension band suture repair has been introduced as an alternative fixation method for certain stable OF types [4, 9, 36]. However, potential advantages of tension band suture repair are still under investigation [36–38] with two multicenter randomized controlled trials currently underway [39, 40].

One of the strengths of this study is the use of the DNPR, which provides nationwide data. In Denmark, public and private hospitals are legally mandated to report data to the DNPR, ensuring comprehensive and reliable data collection [18–20]. Therefore, missing data likely only include patients who did not seek emergency treatment for OFs. We believe this represents a very small group, as healthcare is free in Denmark [19].

A limitation of this study is the lack of validation for the OF diagnosis and surgical procedure codes in the DNPR. However, the overall validation of orthopedic diagnoses and procedures in the DNPR is known to be high [20, 22, 41], and we have no reason to believe that the reporting practices to the register changed during the study period. Moreover, the use of register-based data introduces the risk for misclassification bias, as coding errors or variations in reporting may affect the accuracy of fracture diagnoses and treatment classifications. Additional limitations include the lack of information on patients’ comorbidities, such as osteoporosis and trauma mechanism. Furthermore, we were unable to provide specific details about fracture patterns and severity, which limits the interpretation of treatment decisions, as fracture severity is usually required to determine surgical or non-surgical management.

## Conclusions

The incidence of OFs increased by 29% from 1999 to 2018, with an exponential increase in females ≥ 50 years, likely due to osteoporosis. Non-surgical treatment was predominant across all ages, yet older patients were more likely to be operated than younger patients. The use of PF steadily increased during the study period showing equal numbers for PF and TBW in 2018.

Future research may identify which patients benefit most from surgical or non-surgical treatment by incorporating patient-specific factors such as age, activity level, and comorbidities, thereby refining decision-making and optimizing outcomes that matter to patients.

## Data Availability

Patient data were extracted from the Danish National Patient Register covering a period from 1999 to 2018.

## Abbreviations

OF: Olecranon fracture
TBW: Tension-band wiring
PF: Plate fixation
IR: Incidence rate
DNPR: Danish National Patient Register
ICD-10: International Statistical Classification of Diseases, 10th Revision
NOMESCO: Nordic Medico-Statistical Committee
DASH: Disabilities of the Arm, Shoulder, and Hand
RECORD: Reporting of Studies Conducted Using Observational Routinely-Collected Data
